# Rehabilitation Interventions in Adults with Amyotrophic Lateral Sclerosis: A Review -

**DOI:** 10.31661/gmj.vi.3708

**Published:** 2025-07-02

**Authors:** Maryam Behroozinia, Saeid Khosrawi

**Affiliations:** ^1^ Department of Physical Medicine and Rehabilitation, School of Medicine, Isfahan University of Medical Sciences, Isfahan, Iran

**Keywords:** Amyotrophic Lateral Sclerosis, Rehabilitation, Exercise Therapy

## Abstract

Amyotrophic Lateral Sclerosis (ALS) is the most common and rapidly devastating neurodegenerative disease, which causes impairment of motor neurons in the upper and lower limbs, as well as in the bulbar muscles among adults. This leads to progressive weakness of voluntary muscles. The median survival after the emergence of initial symptoms is typically three years. During this period, due to the worsening of general well-being and independence, patients and their caregivers experience significant emotional stress. Furthermore, there is currently no definitive treatment for ALS. Consequently, patients face various challenges associated with motor impairment, including mobility disturbances, respiratory dysfunction, speech difficulties, and limitations in activities of daily living. Therefore, rehabilitation plays a vital role as a component of multidisciplinary care for managing these issues and reducing the impact of the disease on patients and their families. It is considered the optimal choice for alleviating the discomfort of ALS patients until a curative treatment is discovered.This narrative review aims to provide an overview of different aspects of rehabilitation, including physical therapy, occupational therapy, speech therapy, and respiratory strategies focused on enhancing independence, functional abilities, and overall quality of life while minimizing disabilities and complications in patients coping with this debilitating condition.

## Introduction

Amyotrophic lateral sclerosis (ALS), also called Lou Gehrig’s disease or Charcot disease, is a specific, swiftly progressive, paralytic, lethal disorder characterized by degeneration of upper motor neurons in the brain cortex and lower motor neurons in the brainstem and spinal cord [1][2]. In 1869, Jean-Martin Charcot initially identified this condition as a pure motor neuron disease. However, nowadays, it is considered a neurodegenerative disorder that affects multiple physiological systems [3]. With a global prevalence rate of 4-6 cases per 100,000, ALS stands as the foremost motor neuron disease and ranks third among neurodegenerative disorders, affecting individuals across all racial and ethnic backgrounds. [4][5][6] The majority of ALS patients are between 50 and 65 years old, with only about 5% under 30. The occurrence significantly decreases beyond the age of 80 [7][8]. The typical survival time for ALS patients after symptom onset is about three years [9]. only 5-10% of patients live beyond 10 years after diagnosis [4]. Factors associated with poorer prognosis include an older age when the symptoms first appear, a faster rate of progression at the onset of the disease, and the initial involvement of bulbar muscles [10]. Due to the absence of definitive laboratory tests or imaging biomarkers, the diagnosis is based on clinical manifestations, and an electromyogram can help confirm the diagnosis [11].

The "Revised El Escorial Criteria" have been established for the diagnosis of ALS; the components of these criteria are shown in Table-1 [12]. Based on the Awaji-Shima consensus, ALS has three diagnostic classifications, as shown in Table-2 [13]. Due to its progressive and degenerative nature, ALS affects individuals physically, emotionally, and socially. Rehabilitation is the process of helping disabled patients improve their condition of physical function. It plays a crucial role in supporting ALS patients by addressing the various challenges they face. Because ALS is incurable, by implementing appropriate rehabilitation strategies and clinical support, healthcare professionals can help improve the physical and emotional well-being of ALS patients and extend their survival.

This review aims to provide various rehabilitation strategies, including physical therapy, occupational therapy, speech therapy, and respiratory therapy, that can be employed to improve patient autonomy, functionality, and safety and overall enhance their quality of life while also reducing symptoms associated with the disease. It’s important to note that the specific rehabilitation strategies mentioned in the review will depend on individual patient needs and preferences.

## Overview of Amyotrophic Lateral Sclerosis:

### Pathophysiology of ALS

ALS is classified as sporadic ALS (sALS) with unknown etiology in more than 90% of cases, which do not affect first-degree relatives [14], and the familial form of ALS (fALS), which accounts for nearly 5-10% of cases, characterized by a hereditary pattern. These cases typically appear at an earlier age of onset [15][16].

While the exact reasons for sALS remain unclear, there are various risk factors have been suggested. These factors include age, smoking, low physical fitness, head injuries, cancer history, viral infections (such as poliomyelitis, HHV-6, and HHV-8), as well as occupational and environmental variables such as chemical exposure, pesticide exposure, metal exposure, β-methylamino-L-alanine, and electromagnetic field exposure [17][18].

Recently, several genetic risk factors have been identified for sALS, too; for example, mutations in the C9orf72, SOD1, TARDBP, and FUS genes are associated with both familial and sporadic ALS [19], So it seems that ALS is a multifactorial disease caused by both genetic and various environmental factors [20].

## Clinical Presentation of ALS

**Table:** Table1. Revised El Escorial Criteria for diagnosis of ALS

A-Criteria	1. Clinical, electrophysiological, or neuropathological evidence of LMN degeneration 2. Clinical evidence of UMN degeneration. 3. Progressive spread of symptoms beyond typical nerve supply areas.
B-Criteria	The absence of findings typical of other diseases that could explain the observed symptoms.

The diagnosis of ALS is clinical, and it is confirmed by the presence of the signs of both upper motor neuron (UMN) and lower motor neuron (LMN) in a person with unexplained weakness [12]. UMN disturbance features include spasticity, weakness, and hyperreflexia. By contrast, LMN involvement causes fasciculations, wasting, and weakness [21]. "Split-hand" is one of the characteristic features of ALS that refers to a disproportionate loss of the thenar muscles combined with the first dorsal interossei [22]. ALS has two main clinical manifestations. Limb onset that characterized by MN degeneration signs in limbs and bulbar-onset in about 25-30% of cases characterized by dysarthria, dysphonia, and dysphagia [23][24]. Rarely, atypical presentations, including weight loss, cognitive impairment, behavioral changes, or respiratory failure, are the initial manifestations of ALS [25][26].

During the progression of ALS, the respiratory muscles also weaken, which diminishes respiratory muscle strength, causing an ineffective cough and alveolar hypoventilation as a consequence [27]. Early signs of respiratory involvement include headaches, daytime fatigue, and orthopnea [28]. As respiratory muscle fatigue worsens, patients may experience respiratory failure, often precipitated by pneumonia. Eventually, when patients experience dyspnea at rest, death is imminent [29].

In the late stages of the disease, patients may experience weakness in their axial muscles, which can cause head drop. Additionally, around one-third of patients may experience uncontrollable laughing and crying, referred to as the pseudobulbar effect [30].

Because of weak control of orofacial and Palatino-lingual muscles, facial muscle weakness, and tongue spasticity, excessive drooling, also known as sialorrhea, is considered among the most incapacitating symptoms in ALS [31][32].

Overall, more than 15% of ALS patients have frontotemporal dementia (FTD) [33].

Different factors, such as spasticity, muscle cramps, contractures, and skin pressure, cause pain, which is a common complaint among ALS patients [34].

## Current Treatment Options for ALS

**Table:** Table2. Diagnostic classifications of ALS based on the Awaji-Shima consensus

Clinically definite ALS	· clinical or electrophysiological evidence by the presence of LMN as well as UMN signs in the bulbar region and at least two spinal regions **or** · the presence of LMN and UMN signs in three spinal regions
Clinically probable ALS	· clinical or electrophysiological evidence by LMN and UMN signs in at least two regions with some UMN signs necessarily rostral to (above) the LMN signs
Clinically possible ALS	· clinical or electrophysiological signs of UMN and LMN dysfunction are found in only one region **or** · UMN signs are found alone in two or more regions **or** · LMN signs are found rostral to UMN signs

Despite many research and clinical trials, there is no definitive treatment available for ALS.

Until now, there are two approved treatments for ALS, including Riluzole and Edaravone [35].

Riluzole, an anti-glutamatergic drug that inhibits the presynaptic release of glutamate, is a well-tolerated drug in ALS patients, even in the advanced stage of the disease, which can extend the median survival time by two to three months and enhance the likelihood of survival in the first year by 9% [36][37][38].

It should be taken 50 mg orally every 12 hours. Frequent blood tests to monitor liver function are essential before and during treatment with Riluzole. When the serum levels of transaminases go three times up to normal value, treatment should be stopped [36].

Edaravone (MCI-186) is an antioxidant and a free radical scavenger, which was originally approved as a treatment for cerebral infarction in Japan [39]. It seems to act as a protective agent [40]. The common side effects of it are bruising and disruption in walking. So far, this drug does not have oral administration. Thus, it is administered intravenously to modify ALS clinical outcome [34].

Recently, novel gene therapies targeting potential molecular mechanisms have been developed for the treatment of motor neuron diseases, including ALS. Although regulatory approvals for these therapies are limited, ongoing trials provide hope for effective treatments [41].

## Rehabilitation Interventions for ALS

Rehabilitation for ALS is a comprehensive and multidisciplinary approach. This review delves into the core aspects of rehabilitation in ALS, encompassing Physical Therapy, Occupational Therapy, Speech and Language Therapy, and Respiratory Therapy. Each of these therapies plays a crucial role in addressing the challenges posed by ALS. Physical Therapy focuses on maintaining mobility and preventing complications from muscle weakness. Occupational Therapy aids in adapting daily activities and environments to preserve autonomy. Speech and Language Therapy is essential for communication and swallowing difficulties, while Respiratory Therapy is critical for managing respiratory insufficiency, a common complication in ALS.

## Physical Therapy

**Table:** Table3. Summary of Key Recommendations for Physical Therapy in ALS.

Type of exercise	Recommendations
ROM exercise	Perform 1-2 sessions of active and passive ROM exercises daily for every patient.
Stretching exercise	Done daily at the beginning of the disease
Strengthening Exercises	· Begin muscle strengthening exercises immediately after diagnosis · Mild to moderate level of intensity · Exercise modification should be considered when muscle soreness and fatigue last for more than 30 minutes.
RMT (encompassing IMT and RMT)	· To increase muscle strength: High load and low-speed · To increase muscle endurance: Low load and high-speed

### Stretching and Range of Motion Exercises

Loss of range of motion (ROM) leads to painful adhesive capsulitis and even complex regional pain syndrome [42]. So, ROM exercises are recognized as the initial treatment approach for managing spasticity and alleviating muscle spasms that cause pain in individuals diagnosed with ALS [43].

Although specific recommendations may vary depending on the individual’s stage of the disease, it is generally recommended to perform 1-2 sessions of active and passive ROM exercises daily to enhance or maintain range of motion [44].

ALS patients have weaker muscles, and there is an imbalance between agonist and antagonist muscles, which predisposes them to muscle shortening, joint contracture, and poor postures such as claw hands. Therefore, stretching could enhance flexibility, maintain good alignment of body segments, improve joint mobility, and prevent contractures [45].

Therefore, stretching exercise is a part of standard care for patients with ALS that should be encouraged to be done daily at the beginning of the disease [46]. It can be performed with a caregiver’s assistance when the patient becomes unable to perform stretches independently [47].

### Strengthening Exercises

Resistance exercise improves muscle force/power, induces muscle hypertrophy, maintains skeletal muscle function, and avoids disability [48]. These exercises have a beneficial effect on the quality of life for individuals with ALS; nonetheless, they cannot prolong life expectancy [49]. Studies showed that those who engaged in strength and resistance exercises experienced a lower incidence of falls compared to the group that focused on the range of motion and stretching exercises [50].

It is recommended to begin muscle strengthening exercises immediately after diagnosis, as earlier initiation results in greater improvement. Continuation of these exercises is also an important factor, as a positive effect can be observed approximately a year after the initiation of exercises [51].

The exercise plan should focus on muscles that demonstrate a strength level above three on the Manual Muscle Testing scale and patients should closely be monitored for signs of overuse during these exercises [52]. Because some studies showed that high-intensity resistance training may increase the risk or exacerbate the progression of ALS [53][54]. This happens because of oxidative stress, glutamate excitotoxicity, and heightened calcium loads, which promote selective degeneration of susceptible motor neurons [55][56]. Muscle soreness and fatigue lasting over 30 minutes post-exercise are the signs of overuse and indicate the necessity of exercise plan modification [57].

### Respiratory Muscles Training

Involvement of respiratory muscles leads to decreased subglottic air pressure, ability to cough and clear secretions and consequently increases the risk of pulmonary infection [58][59].

The forced vital capacity (FVC) test measures the amount of air a person can forcefully exhale after taking a deep breath. It is closely associated with both disease progression and survival in ALS patients. When FVC falls below 50%, it indicates the beginning of respiratory failure, which is a critical stage in the disease [60].

Respiratory muscle training (RMT) in patients with ALS can be used as an adjunctive therapy that improves ventilator function and respiratory strength [61]. RMT can be divided into two types: training for the muscles involved in inhalation, known as inspiratory muscle training (IMT), and training for the muscles involved in exhalation, known as expiratory muscle training (EMT). The efficacy of EMT is less clear than IMT [62]. However, prior studies showed that it is an effective tool for improving maximal Peak cough flow (PCF) in individuals with neuromuscular diseases, and 5-week training improves respiratory and bulbar function in individuals with ALS [62][63]. RMT can also have a positive effect on respiratory muscle endurance. While high load and low-speed training will increase muscle strength, engaging in high-speed training with low resistance enhances endurance [64][65].

RMT protocols should consider 4 components known as FITT (Frequency, Intensity, Time, and Type). However, the optimal FITT parameters for ALS-specific RMT remain undefined, underscoring the need for further research to establish effective respiratory management guidelines [66].

POWERbreathe® is a device utilized for the training of inspiratory muscles. When used as a supplementary therapy alongside standard care in neuromuscular conditions, this device provides advantages such as improving the strength of inspiratory muscles and lowering resting heart rate [67][68].

Table-3 provides a summary of physical therapy recommendations in ALS. Overall, although there is no definite evidence, exercise appears to be one of the few modalities that may enhance function in ALS patients. In addition to improving function, it may also provide some control over the disease.

## Occupational Therapy

### Activities of Daily Living Training

Activities of daily living (ADL) training is a crucial component of rehabilitation in ALS patients. It focuses on assisting individuals with maintaining as much independence as possible in performing everyday tasks that are essential for self-care and daily living. Unfortunately, few studies have been done on ADL disability in ALS, but A large variety of adaptive devices are accessible to help them perform ADLs, although no single device is suitable for all patients or all stages of the illness.

Mobility is essential for performing ADLs. ALS can lead to gait alterations and subsequently cause decreased mobility, which in turn increases the risk of falls and fractures. It has been reported that approximately 33% of ALS patients experience falls [69]. Therefore, training may involve techniques and assistive devices to facilitate safe transfers from one position to another.

Various types of mobility aids, including canes, crutches, and walkers, are available for ALS patients. The prescription of each aid is contingent upon the extent of weakness in both the upper and lower limbs, as well as the grip strength. Canes can serve as valuable aids for individuals experiencing gait instability due to weakness. If hand weakness is a concern, canes with a horizontal grip can be particularly beneficial [70]. However, crutches may not be a suitable option in these cases, as their use requires a high level of strength and coordination in the upper limbs. Walkers offer substantial support and are recommended for individuals with moderate to severe balance issues. 4-wheeled walkers, in contrast to standard ones, provide the advantage of not needing to be lifted. However, it’s essential to ensure that the patient can maneuver it safely [46]. Wheelchairs (manual or electric), mobility scooters, and stair lift systems are other examples of products that could be used to facilitate transportation indoors and outdoors [71]. Orthotic devices such as ankle-foot orthoses, night splints, wrist extension orthoses, thumb positioning orthoses, and cervical collars are also employed to aid in enhancing function and mobility among individuals with ALS [72].

One part of basic ADLs includes personal care such as hygiene or grooming, dressing, and toileting. Occupational therapists can evaluate the needs of patients and provide equipment to enhance independence and function. This may include specialized utensils to improve grip for self-feeding, raised toilet seats, grab bars, shower chairs, and transfer devices for bathing [73].

### Energy Conservation Strategies

One of the frequent symptoms of ALS is fatigue (in approximately 44-86% of cases), which consequently could decrease quality of life [74]. Therefore, knowing strategies for diminishing this symptom is one the crucial parts of the management of ALS patients.

Some energy conservation strategies include sufficient rest periods throughout the day, having proper posture during activities, for example, performing tasks while sitting rather than standing, splitting fatiguing activities into several smaller and easier components, prioritizing activities emphasizing doing hard functions at the beginning, teaching ergonomic principles, and environment modification. These techniques usually are instructed by the occupational therapist [75][76][77][78].

Among the energy conservation strategies mentioned, the most commonly used and effective ones were those incorporating periods of rest, particularly those that maintained a balance between work and rest [79].

Certain assistive devices consume more energy than others. For instance, a rolling walker requires less effort compared to a standard walker. Similarly, single canes are more energy efficient than crutches or quad canes. Another factor that can influence energy expenditure is the material composition of different assistive devices utilized by patients. For instance, a lightweight carbon fiber ankle-foot orthosis is generally preferred over heavier hinged ones [80].

There are also some simple conserve energy strategies that can be helpful for ALS patients while they want to speak. First, they should avoid any noisy situation, for example, by muting TV or asking others to talk to them in a quiet situation instead of a crowded room. Also, they can use a voice amplifier to reduce effort [81][82].

## Speech and Language Therapy

### Augmentative and Alternative Communication

ALS can significantly limit a person’s ability to communicate a few years after the initiation. In a way, more than 80% of patients experience difficulties with their speech and communication, and most will be unable to speak at all [83]. Recognizing this impairment is crucial because a lack of effective communication leads them to prevent engaging in various activities, resulting in social isolation, which significantly diminishes their quality of life [84].

Thus, augmentative and alternative communication (AAC) is becoming increasingly important, offering ALS patients a valuable tool to overcome the significant motor impairments they face. They also can help patients to keep their emotional connection with other people, reducing the load on caregivers and enhancing the psychological and social well-being of the patient and QOL in dysarthric ALS patients, despite lack of speech intelligibility [85]. In addition, using AAC strategies can diminish patients ‘anger and frustration experienced by individuals who have lost the ability to communicate with others using natural speech [86].

It should be kept in mind that appreciate timing for referring patients to AAC assessment and intervention is an important decision-making issue [87].

Strengthening exercise of lip and tongue may sometimes be helpful for patients to pronounce words more clearly but so far, there hasn’t been any evidence that orofacial muscle strengthening could be effective [88].

ALS patients may use non-verbal strategies for communication, which means that they send their messages by gesture, facial expression, or eye contact [86].

As long as patients ‘hand function is adequate, writing can be used instead of speaking. Devices needed are very simple, for example, paper and pencil, alphabet cards, a portable typewriter, and a letter board. These are the examples of low-tech ACC. For those who cannot rely on speaking or writing, then high-tech AAC such as brain-computer interface (BCI) and eye tracking systems should be considered as a part of palliative care [89][90].

BCI is one of the usable devices for disabled patients with ALS, which interprets the electric signals of the brain and translates them into commands. The BCI has a virtual keyboard known as a P300 speller [91]. This virtual keyboard includes a word prediction feature that facilitates word completion and next-word prediction. It accomplishes this by presenting a list of the ten most probable words on the right side of the keyboard. One of the best features of this device is its easy operation, which does not require much learning effort [92].

For individuals whose mobility is limited to their eyes, eye-tracking-based applications could be useful, which enable them to communicate with normal people and make their lives easier [93]. A former study indicated that the worse the clinical presentation, the better acceptance was achieved by the patients [94]. As it is obvious from its mechanism of action, the necessary factor for using this technology is full ocular mobility and having appropriate visuals [95]. Fortunately, eye gaze remains intact over time, even in cases where invasive ventilation has been used [96].

### Dysphagia Management

About 85% of people with ALS encounter dysphagia, or swallowing impairment, leading to aspiration and malnutrition, negative prognostic components in ALS, which increase the mortality rate by 7.7 times, Therefore, when oral intake becomes insufficient, excessively challenging, or poses safety risks, it is necessary to use alternative feeding methods for adequate nutrition to stabilize body weight [97][98][99].

In the initial stages of dysphagia, dietary modification including soft, semi-solid, and semi-liquid foods and some swallowing techniques such as supraglottic swallow (taking a deep breath and holding it during the swallow, and coughing immediately after swallowing), chin-tuck maneuver (pushing the base of the tongue toward the pharyngeal wall) and regulating bolus sizes can be helpful [100][101].

High-energy supplementation with no adverse effect could stabilize body weight. Thus, the use of high-energy supplementation is suggested [102].

A reduction in body weight exceeding 5%-10% of an individual's typical weight, a BMI <20 kg/m², and dysphagia are indications for considering feeding tube placement [103]. It is important to note that enteral feeding does not need to replace oral feeding entirely and can be used to supplement oral intake.

Nasogastric tube feedings can be used for nutrition support, but they are not recommended for long-term use because they increase the risk of aspiration and are uncomfortable for patients. Also, nasogastric tubes increase oropharyngeal secretions so that they may cause ulceration [104][105].

For most patients, endoscopic placement of a percutaneous gastrostomy (PEG) is appropriate. Patients should be referred for this procedure before their FVC decreases to 50% of the predicted value (measured in a seated position) [106], as declining FVC is associated with increased morbidity and mortality during tube placement [107].

An alternative for PEG is PRG (percutaneous radiologic gastrostomy), which can be more tolerated than PEG, especially for patients with respiratory failure, because, unlike PEG, it doesn't need any sedation, but it's not as available as PEG, and the likely of leakage and displacement is higher [108][109].

Table-4 provides a summary of dysphagia management strategies. Overall, effective dysphagia management in ALS requires a multidisciplinary approach that includes dietary modifications, safe swallowing techniques, nutritional support, and the timely initiation of enteral feeding when indicated. Early intervention and continuous monitoring are crucial to preventing complications associated with dysphagia, thereby improving the overall quality of life for ALS patients.

## Respiratory Therapy

**Table:** Table4. Summary of Key Recommendations for the Management of Dysphagia

Dietary modification, including soft, semi-solid, and semi-liquid foods and regulating bolus sizes
Swallowing techniques such as supraglottic swallow and chin-tuck maneuver
High-energy supplementations
Feeding tube placement when: · body weight exceeding 5%-10% of an individual's typical weight · BMI <20 kg/m² · dysphagia Feeding tube options include: · Nasogastric tube: Suitable for short-term use. · PEG: Recommended before FVC decreases to 50% of the predicted value. · PRG: Indicated for patients with respiratory failure, particularly those with an FVC of less than 50%.

**Table:** Table5. Summary of Key Recommendations for the Management of Respiratory Care in ALS

Cough Assist Techniques	· Breath-stacking technique · Manual assist technique · High-frequency chest wall oscillation · Mechanical Insufflation-Exsufflation
NIV	Indication: · Maximum inspiratory pressure ≤ 60 cm H_2_O, · Spo2≤ 88% for at least five continuous minutes · Paco2> 45 mm Hg · Symptoms related to hypercapnia
NIPPV	Indication: · FVC is less than 50% of the predicted value Titration: · Starting at 8 cm of H_2_0 IPAP and 4 cm of H_2_0 EPAP
Invasive Ventilation	Indication: · When NIV is not well-tolerated · NIV for more than 18 hours per day

### Noninvasive Ventilation

Many ALS patients, despite mild or no respiratory symptoms, have abnormal FVC at presentation [58]. For this reason, assessment of respiratory insufficiency, including symptoms of nocturnal hypoventilation such as daytime headache and sleep disturbance, should be done at every visit [110].

Non-invasive ventilation (NIV) is applying positive pressure to the airway through any device other than the endotracheal tube [111]. This therapy in ALS patients is associated with increasing QOL and survival despite disease progression, especially in individuals with orthopnea, without having any adverse effect on caregivers'QOL or increasing their stress [112][113]. Also, it can decrease energy consumption in ALS patients [114].

Maximum inspiratory pressure ≤ 60 cm H2O, Spo2≤ 88% for at least five continuous minutes, Paco2> 45 mm Hg, and symptoms related to hypercapnia are indications of NIV in patients with ALS [58][115].

NIV can be delivered through different methods, including nasal masks, oronasal masks, full-face masks, and mouthpieces. In cases of chronic respiratory failure, nasal masks are used, while oronasal masks are used in emergency situations due to their ability to minimize leakage and improve the effectiveness of ventilation [116]. For patients who suffer from skin lesions, the total face mask is a possible alternative option [117]. The other way for NIV is through mouthpieces, which may be considered for patients who experience difficulty tolerating oronasal and nasal masks. Also, it can be beneficial for individuals who encounter issues such as skin lesions, eye irritation, or gastric irritation [118].

It is crucial to consider that oxygen should not be used instead of NIV because ALS patients suffer from hypoventilation with hypercapnia, not hypoxia [119].

NIPPV (noninvasive positive pressure ventilation) is currently recommended to start when FVC is less than 50% of the predicted value [120]. So far, there is no optimal titration for NIPPV pressure, but U.S. ALS clinic medical directors recommended starting this therapy at 8 cm of H20 inspiratory (IPAP) and 4 cm of H20 expiratory positive airway pressure (EPAP) [121].

The ones who use NIPPV should be monitored through pulse oximetry overnight and regular appointments with a respiratory physician should be scheduled to confirm that the pressure settings are appropriate [122].

### Cough Assist Techniques

ALS is associated with a reduction of cough flow and efficacy because inspiratory muscle weakness leads to a lower depth of pre-cough inspiration, and expiratory muscle weakness decreases intrathoracic expiratory pressure [123]. Therefore, the inability to have an effective cough is one of the major problems faced by most ALS patients.

One of the first-line and low-cost strategies to improve airway clearance and cough augmentation in ALS patients is the breath-stacking technique; in this technique, a manual resuscitation bag with a one-way valve is used to deliver a large breath volume to the patient's lungs [63].

The manual assist technique is another cough assist technique for ALS patients, which requires a trained person to apply pressure on the patient's chest with one forearm while the other hand thrust on the abdomen during expiration [124].

Neurodegenerative disease also leads to difficulty in clearing secretions because of respiratory muscle weakness and bulbar insufficiency. High-frequency chest wall oscillation by using a wearable vest helps to mobilize secretions and decrease symptoms of breathlessness in these patients. In three studies, it was used twice a day for 10-30 minutes per session [125][126]. If secretions are tenacious or purulent, chest physiotherapy with percussion could be useful [127].

Mechanical Insufflation-Exsufflation (MI-E) is a therapy designed to generate effective expiratory flow rates in medically stable patients with bulbar and nonbulbar ALS [128]. The recommended initial MI-E settings typically include an inspiratory pressure of +40 cm H2O and an expiratory pressure of -40 cm H2O. However, adjustments may be required based on individual patient factors, such as height and lung capacity, to minimize the risk of complications, including pneumothorax [129].

### Invasive Ventilation

Individuals who experience bulbar dysfunction and excessive sialorrhea may not tolerate non-invasive ventilation [27]. In situations where non-invasive ventilation is not well-tolerated or becomes insufficient due to advancing weakness of the respiratory muscles, an alternative option is invasive ventilation, specifically tracheostomy. This involves the insertion of a tube into the lower respiratory tract to provide assisted breathing support [130][131]. It is also performed in patients on continuous NIV for more than 18 hours per day [132].

Tracheostomy provides better ventilator pressure and gas exchange due to less air leakage in comparison with NIV. However, it can lead to a decline in patients' physical function and significantly increase the burden on caregivers [133][134]. 70% of patients experience loss of swallowing and limb function after tracheostomy [135]. There is a marked difference in the prevalence of tracheostomy in different geographic regions, even within areas of the same country [132]. One explanation for this variation is social, cultural, and religious differences, which affect the decision-making process for introducing tracheostomy [136].

The decision to stop tracheostomy varies among different countries, but mostly it is legal [137].

Among the various respiratory treatments for ALS, there is an experimental strategy called acute intermittent hypoxia. This method involves repeated brief reductions in inspired oxygen levels (~9-10% oxygen) for 60 to 90 seconds, separated by intervals of normoxia (21% oxygen) for 1-2 minutes. It may have the potential to preserve breathing function in ALS patients [138]. Overall, effective management of respiratory complications in ALS is vital for improving patient outcomes and quality of life. Integrating treatments such as non-invasive ventilation (NIV), cough-assist techniques, strength training, and, when indicated, invasive ventilation should be considered an essential part of comprehensive care for these patients. Table-5 summarizes respiratory therapy management strategies.

## Challenges and Barriers to Rehabilitation Interventions for ALS

### Patients Factors

Rehabilitation interventions pose various challenges for individuals with ALS. One of the most challenging issues for patients dealing with ALS is financial constraints because the cost of rehabilitation services, assistive devices, and medications can be a significant barrier for them; as Henschke et al. indicated in their study, many ALS patients in Germany commonly face issues with the availability and financing of assistive devices [139].

Another obstacle arises for ALS patients residing in remote and rural areas who face difficulty accessing well-equipped multidisciplinary clinics. Additionally, although technological advancements in ALS rehabilitation a good news, it is important to consider that some patients, particularly the elderly, may encounter challenges in adapting to certain technologies, so they need additional time for learning.

### Caregiver Factors

Primary caregivers for patients with ALS are usually family members who face many challenges. The most common challenge for them has been reported travel and the cost of treatments. As a previous study indicated, in addition to the stress of leaving a family member, caregivers also grapple with the fear of depleting their savings on medical expenses [140].

Many of them find it difficult to balance their time with their caregiving responsibilities, also they may suffer from psychosocial distress [141]. Caregivers neglect their own needs to take care of the person with ALS, which can threaten their well-being [142]. A study demonstrated that increased behavioral and physical impairment in ALS patients is linked to a higher prevalence of depressive feelings among caregivers [143].

### Healthcare System Factors

A multidisciplinary team for patients with ALS consists of a physiatrist, neurologist, gastroenterologist, social worker, occupational therapist, speech therapist, pulmonologist, nurse, physiatrist, dietitian, and psychologist [144]. To achieve the optimum results from rehabilitation, comprehensive and coordinated care among these specialists and health care is needed, so lack of integration of care across different providers can be challenging [145].

Another healthcare barrier that hinders effective ALS rehabilitation is that limited awareness and understanding of ALS among healthcare professionals may cause delays in diagnosis and inadequate referrals to rehabilitation services [146]. It appears that more than half of the patients received alternative diagnoses rather than a confirmed diagnosis of ALS [147]. Thus, ongoing education and training for healthcare providers are essential to improve ALS care and rehabilitation.

One of the restrictions of healthcare systems for the rehabilitation of ALS patients is that they may face difficulties in terms of resources, including the availability of specialized equipment, assistive devices, and access to rehabilitation facilities. Also, Insurance coverage can limit ALS patients' ability to access needed rehabilitation interventions, assistive technologies, and support services.

It's important to consider that these barriers and challenges can vary across different regions.

## Conclusion

Currently, since there is no cure-all for ALS, treatment mainly targets managing symptoms. Yet, certain therapies show promise in slowing its progression and potentially enhancing survival rates. ALS profoundly affects individuals, significantly impairing motor abilities, communication, and sometimes even nutrition and breathing. This makes rehabilitation a critical part of all-encompassing care for those afflicted.

In our paper, we've aimed to explore various rehabilitation aspects for ALS patients. However, it's clear that research in this area is lacking, and there's a noticeable absence of comprehensive guidelines.

Future efforts need to focus on developing groundbreaking rehab strategies and fostering stronger teamwork across various medical disciplines. As research progresses, we anticipate the ALS rehabilitation field will evolve, offering enhanced support to patients and their families.

## Conflicts of Interest

There is nothing to declare.
